# Improved Effort and Cost Estimation Model Using Artificial Neural Networks and Taguchi Method with Different Activation Functions

**DOI:** 10.3390/e23070854

**Published:** 2021-07-02

**Authors:** Nevena Rankovic, Dragica Rankovic, Mirjana Ivanovic, Ljubomir Lazic

**Affiliations:** 1 School of Computing, Union University, 11000 Belgrade, Serbia; drankovic@raf.rs (D.R.); ljlazic@raf.rs (L.L.); 2 Faculty of Sciences, University of Novi Sad, 21000 Novi Sad, Serbia; mira@dmi.uns.ac.rs

**Keywords:** software development estimation, artificial neural network design, orthogonal array-based experiment, clustering, fuzzification, activation function choices

## Abstract

Software estimation involves meeting a huge number of different requirements, such as resource allocation, cost estimation, effort estimation, time estimation, and the changing demands of software product customers. Numerous estimation models try to solve these problems. In our experiment, a clustering method of input values to mitigate the heterogeneous nature of selected projects was used. Additionally, homogeneity of the data was achieved with the fuzzification method, and we proposed two different activation functions inside a hidden layer, during the construction of artificial neural networks (ANNs). In this research, we present an experiment that uses two different architectures of ANNs, based on Taguchi’s orthogonal vector plans, to satisfy the set conditions, with additional methods and criteria for validation of the proposed model, in this approach. The aim of this paper is the comparative analysis of the obtained results of mean magnitude relative error (MMRE) values. At the same time, our goal is also to find a relatively simple architecture that minimizes the error value while covering a wide range of different software projects. For this purpose, six different datasets are divided into four chosen clusters. The obtained results show that the estimation of diverse projects by dividing them into clusters can contribute to an efficient, reliable, and accurate software product assessment. The contribution of this paper is in the discovered solution that enables the execution of a small number of iterations, which reduces the execution time and achieves the minimum error.

## 1. Introduction

In recent years, due to a significant evolution in adopting and developing new technologies and methodologies in the area of software effort estimation, many researchers are attempting to optimize the accuracy of this process [1,2]. Since this is one of the crucial processes in completing a software product, there is a continuous need for questioning both overestimation and underestimation [3]. We can relate costs to the project in the initial phase, depending upon the efforts needed [4,5]. This involves a large number of efficiency reviews using different techniques. Two essential types of estimation techniques, like model-based and expert-based techniques, have been introduced.

The first one has a foundation depending on mathematical models, while the second one has accepted human guidance [6]. Nowadays, estimation has turned attention to various machine-learning (ML) methods and hybrid approaches that combine parametric and non-parametric models [7,8,9]. Researchers use different higher-order ANN architectures and deep-learning networks to combine parametric models such as COCOMO, FP, and UCP for use in software effort estimation. This shift of interest occurred because of the preponderance of traditional approaches focusing on particular aspects of the process of development, while ignoring others. Additionally, some of these adjustment factors might be affected by specific rules, work environments, or even different cultures [10].

The essential part of using artificial intelligence (AI) in software effort estimation is the construction of the Artificial Neural Network (ANN) architecture [11]. ANNs have learning adaptability and are good at modeling complex nonlinear relationships. They are also flexible in incorporating expert knowledge into the prediction model. The software market proposes many software cost estimation models developed using ANNs over the years [12,13].

New approaches founded on ANN architecture have disadvantages that indicate the optimal set of parameters during training, when achieving the convergence rate and the required accuracy. This results in a vast number of iterations that need to be performed. In this paper, we propose two different ANN architectures, based on the Taguchi method for robust design which uses three software development attributes in COCOMO (constructive cost model) models as control variables (scale factors, cost factors, and software size) [14]. COCOMO is an algorithmic cost model where, with the help of mathematical functions, a context between software metrics and project costs is created. Actual effort is the real value of a project based on the number of lines of code expressed in person-months (PM).

The critical decisions that defined the objective of our research are as follows: (1) a comparison of the two activation functions within the hidden layer of the ANN architectures, in order to reduce the number of iterations required to assess efforts to develop software projects; (2) a comparison of the obtained MMRE results, depending on the complexity of the proposed architecture; (3) finding one of the most efficient methods of encoding and decoding input values, such as the fuzzification method; (4) ascertaining the minimum number of iterations to be performed, i.e., shortened estimation time; (5) the division of data sets into clusters according to the actual effort, to mitigate the heterogeneous nature of the projects; (6) the testing and validation of different data sets in order to prevent errors and confirm results.

In our approach, we introduce six different datasets in three phases of the experiment using fuzzification [15,16,17] and the clustering method, to prove the reliability and accuracy of our model. To homogenize the heterogeneous nature of the projects, we divide the input signals using four different clusters, according to their actual efficiency. The Taguchi method includes orthogonal vector arrays that are explicitly ordered in the matrix [18,19]. This method allows the development of a prediction method that is less complex and much more useful. We use a back-propagation neural network [20] and relate it with the two different orthogonal arrays (OA). The number of parameters equals the number of weights of the ANN. The interval containing the solution is gradually dwindling, as the result of an iterative robust design algorithm. The numerical value of the cost-effect function that is observed in every examination in data distribution is sensitive to individual predictions [1,3]. To increase the stability and safety of the experiment, prediction and correlation between the estimated and actual values are calculated. Metrics to monitor progress, such as the mean absolute error (MAE), and mean magnitude relative error (MMRE), were used. Besides reducing the values, we compare the obtained MMRE results with two different activation functions like the sigmoid function and hyperbolic tangent function. Finally, to confirm our experiment’s and functions’ obtained values and efficiency, two different ANN architectures and activation functions are compared.

The motivation of our research is to achieve:

- A simple ANN architecture that requires a short training time (small number of iterations, i.e., epoch, which is less than 10). Acceptable error for application in other areas with a critical mission, where a rapid response time to the studied problem is required;
- A stable training process of ANN architecture, no oscillations, peaks, etc.;
- A reliable ANN architecture training process that has been validated on a large number of datasets from practice;
- The minimization of the required number of observations in order to quickly and effectively train the ANN architecture;
- The prediction accuracy required to be the same or better than other approaches (COCOMO, function point analysis, use case point analysis, etc.), which can be compared to estimating the magnitude of software development efforts.

The outcome of our research is as follows:

- The method of clustering achieved homogenization of the heterogeneous nature of the projects of each used dataset;
- The MMRE value is in the range of 30.1% to 48.2% for the ANN-L27 architecture, depending on the nature of the projects of the dataset used;
- The MMRE value is in the range of 31.1% to 49.2% for the ANN-L36 architecture, depending on the nature of the projects of the dataset used;
- The best results are achieved with the sigmoid activation function of the hidden layer and the output layer;
- The number of iterations performed is in the range of 5 to 9, depending on the given dataset and the cluster within the dataset;
- The model’s efficiency, reliability, and accuracy were confirmed through two correlation coefficients (Pearson’s and Spearman’s);
- Additional prediction monitoring found that the ANN-L36 architecture was about 1% better.

Our proposed approach can be applied to the implementation of software projects in the field of software engineering, as well as in other areas such as medicine, nuclear sciences, speech recognition, solar systems, neuroscience, and others [21,22,23].

This paper is structured as follows: Section 2 provides an overview of the latest research in the area of software estimation using various ML algorithms and ANNs. Section 3 describes the proposed approach to software development estimation. It is divided into three parts: the first part presents a robust design technique based on Taguchi orthogonal arrays, the second part presents information on the dataset used, and the third part describes the methodology used in detail. The results obtained are discussed in Section 4, while concluding remarks and ideas for future work are given in Section 5.

## 2. Related Work

One of the riskier projects in the modern industrial arena is developing software projects [24]. Customer requirements, methodology, tools, and the intangible nature of the products can significantly impact the project schedule, the quality of the software product, estimated effort, and related costs. In this manner, the risk of successful completion of the project can be understood as an event that could have the consequences of a particular hazard [25,26,27].

Many studies state that risk assessment in software project planning is rarely found and is often challenging to implement [28,29]. Machine learning (ML), as one of the branches of artificial intelligence, has gained importance in the field of software assessment. The study [6] optimizes the estimation of costs in software development, using the COCOMO 2000 and applying two different algorithms on presented models, like the dolphin algorithm and the hybrid dolphin and bat algorithm (DolBat). The results show a lower value of magnitude relative error (MRE). However, in our approach, we show that it is possible to reduce this MRE value, i.e., to obtain better results with a relatively simple ANN architecture to perform a minimum number of iterations, reducing the estimation time.

Research by [20,30] represents the use of a combined technique, as they combine an application of ML that improves the COCOMO model using ANNs; however, the results achieved for MRE were again relatively high. An interesting study was conducted on different neural network algorithms, and their comparison to accurately estimate software costs. This study also used other activation functions that gave worse results than the sigmoid function [31].

Contrary to our approach, the speed of convergence in their work is much slower. In other studies, like that in [32], a two-layer network was used as an improved model to minimize the MRE value between actual costs and estimated costs.

The research model of a multilayer neural network for estimating software effort in real-world applications [33,34,35], with the function of activating identity on the input layer, hidden layer, and the output layer, achieved a slightly better result. However, a smaller number of performed iterations is not achieved.

Contrary to our approach, other researchers, for example in the study by [36], analyze different feature selection algorithms to increase the accuracy of software development effort predictions. Moreover, they investigate both bio- and non-bio-inspired algorithms. In comparison, the authors in the study by [37] focus on comparing the stochastic regression models with the proposed gradient boosting regressor model.

Based on several experiments, we conclude that a more complex architecture gives a lower value of MMRE. By introducing a new, more complex ANN-L36 architecture, we tried to reduce the value of MMRE, compared to the ANN-L27 architecture. In addition to the clustering method, which in our previous study was limited to three selected clusters, in this experiment, we increased the granularity by dividing the datasets into four clusters and introducing the fuzzification as well as defuzzification methods. The heterogeneous nature of the projects is further mitigated and controlled by these methods. In addition, a comparison with another activation function in the hidden layer, such as the hyperbolic tangent, was discussed. In this approach, the number of required iterations is further reduced, which results in shortening the time required for fast and accurate estimation because the convergence rate of the two proposed ANN architectures is less than 8. Monitoring the correlation coefficients and prediction on three different criteria confirmed the efficiency of the proposed approach.

## 3. Proposed Approach

The ANN network architecture includes input values, none or more hidden layers, and an output layer. The shape, type, and size of ANN training parameters affect the specific ANN architecture. In constructing all the proposed architectures in this paper, the Taguchi orthogonal array was used to optimize the design parameters. In ANN design that uses the Taguchi methodology, the engineer must recognize the application problem well. The advantage of this approach over other nonlinear models is based on estimating any function with optional precision. To simplify optimization problems, this paper uses various Taguchi orthogonal arrays representing the MFFN (multilayer feed-forward neural network) class, which has a vital role in solving various types of problems in science, engineering, and engineering medicine, pattern recognition, nuclear sciences, and other fields [1]. In order to construct a high-performance MFFN, no clearly defined theory allows the calculation of ideal parameter settings. This leads to the conclusion that even small changes in parameters can cause significant differences in the behavior of almost all networks. In [2], an analysis of neural network design factors and object functions is given, in which an architecture with one or two hidden layers is recommended. Based on the Kolmogorov–Smirnov test, a recommendation is given that the number of neurons in the hidden layer should be twice the number of input neurons, increased by one, i.e., N-input + 1. The results for each of the 240 experiments were collected. The authors showed that a specific neural network configuration is required to achieve convergence, along with the accuracy of the trained network, when a set of test data is obtained. They also concluded that the number of hidden layers (one or two) has a minimal effect on the network accuracy but is rather significant at the convergence speed. Considering these results, we adopted a trial-and-error strategy, because most existing theoretical works for generalization fail to explain the performance of neural networks in practice. There are many different domains where various AI methods have been applied as solution approaches, such as online learning, scheduling, multi-objective optimization, vehicle routing, medicine, data classification, and others (not only software development estimation) [38,39,40,41,42,43].

**Figure 1:**
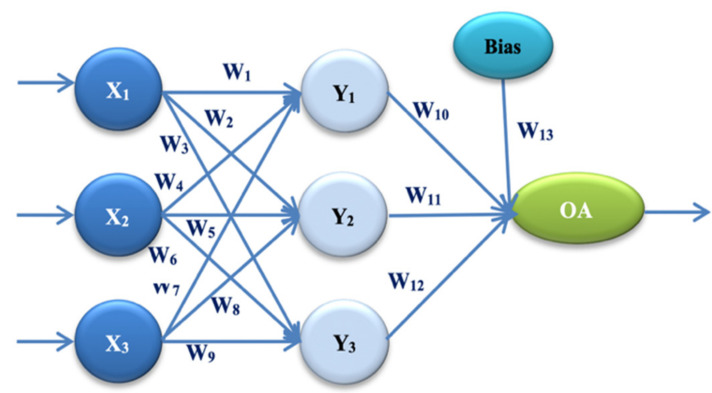
ANN architecture with one hidden layer (ANN-L27).

**Figure 2:**
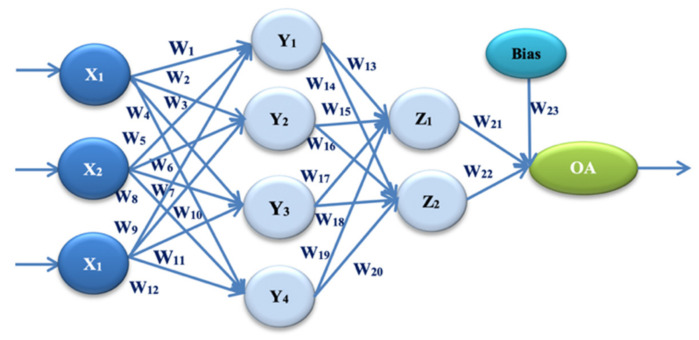
ANN architecture with one hidden layer (ANN-L36).

In our experiment, two different architectures of artificial neural networks were proposed. These architectures are based on two different Taguchi orthogonal plan vectors. The architecture of the artificial neural network is established on the orthogonal array L27, with 13 weight parameters. It consists of one input layer, one hidden layer, and one output layer. The input layer has three input values (signals): X1 = E, X2 = PEMi, and X3 = KLOC. By combining the input values and weight parameters using the appropriate activation function, the obtained values are: Y1, Y2, and Y3 in the hidden layer. One output size—the estimated effort—is calculated by combining the sizes from the hidden layer and the remaining weight parameters. OA denotes the output value in Figure 1. Later in the paper, this is referred to as ANN-L27. The architecture of the artificial neural network is established on the orthogonal array L36, with 23 weight parameters. It consists of one input layer, two hidden layers, and one output layer. The input layer has three input values (signals): X1 = E, X2 = PEMi, and X3 = KLOC. By combining the input values and weight coefficients with the appropriate activation function, the values are obtained: Y1, Y2, Y3, and Y4 in the first hidden layer. Two values are calculated in the second hidden layer, Z1 and Z2, by combining the values from the first hidden layer with the corresponding weight parameters. One output value—estimated effort—is calculated by combining two values with weight parameters from the second hidden layer. OA denotes the output value in Figure 2, later in the paper referred to as ANN-L36. After several trial experiments, those clustering and fuzzification methods that gave the best results were selected. The method that divides the input signals into four clusters according to the value of the actual effort has made it possible to lower the error and control the heterogeneous values of the selected projects. The fuzzification method further homogenizes the data to reduce the number of iterations as much as possible. After all three phases of the experiment (training, testing and validation of the two proposed ANN architectures), to determine the validity, reliability, and efficiency of the proposed models, the prediction and correlation of all six used datasets were monitored.

### 3.1. Robust Design Technique—Taguchi Orthogonal Arrays

The first proposed ANN architecture in our experiment is ANN-L27. This architecture is based on the Taguchi orthogonal array L27 with 13 parameters [1,2,3] (W_i_, *I* = 1, …, 13), and three levels: L1, L2, and L3 (Figure 1, Table 1).

The second proposed ANN architecture in our experiment is ANN-L36. This architecture is based on the Taguchi Orthogonal Array L36, with 23 parameters [1,2,3] (W_i_, *I* = 1, …, 23). It consists of two parts. The first part has 11 parameters on two levels, L1 and L2, while the second part has 12 parameters on three levels, L1, L2, and L3 (Figure 2, Table 2).

### 3.2. Used Dataset

Through all phases of the experiment, both ANN-L27 and ANN-L36 were used. For the first phase of our training experiment, 87 projects from the COCOMO 2000 dataset were chosen. Table 3 shows that the 23 projects from the COCOMO 2000 were used in the testing phase. Once again, to check and confirm the obtained results, four datasets for the third phase of our experiment were used. In the first validation, randomized 46 selected projects from the COCOMO 81 dataset were used. In the second validation, 60 projects from the NASA dataset were used. Finally, in the third validation, the Kemerer dataset with a dataset of industrial projects (15 + 5) was used. Finally, in the fourth validation, a Desharnais dataset of 80 projects was used. Basic information on the datasets used is shown in Table 3. Within Dataset_1, the data range is from minimum actual effort values from 8.4 to maximum actual effort values of 8211, expressed in person-months [PM]. In all other phases of the experiment, datasets that have project values within this scope were used. This infers taking projects of different scales that require different efforts to implement them, as shown in Table 4. Data sets are selected depending on input sizes. In this paper, input sizes are the determining factor for selecting datasets. In the paper [44], the authors showed that the R^2^ value for COCOMO is 0.7203 and, for example, for the Function Point is 0.4392, which is additional proof that our approach is statistically reliable. In our paper, this is confirmed with the Pearson’s and Spearman’s rho correlation coefficients. Furthermore, the paper published in Elsevier [7] presented the analysis about ISBSG-10 dataset usage, and the results tell us that only one of 80 authors used this dataset for COCOMO-based attributes as software efforts predictors. The authors in the paper [45] did not consider the ISBSG, which is the most used one. Based on the correlation values, we choose the appropriate datasets [6,20,46]. The results in Table 4. indicate the heterogeneous nature of the designs of each dataset used and directly affect the prediction results within all three phases/parts of the experiment (training, testing, validation). It can be seen that the data sets from 1 to 6 are very heterogeneous in terms of the programming languages used, the duration of application development, and an extensive range of actual effort values, with a large standard deviation. Datasets used in this experiment are publicly available at [47].

### 3.3. Applied Methodology

**Figure 3:**
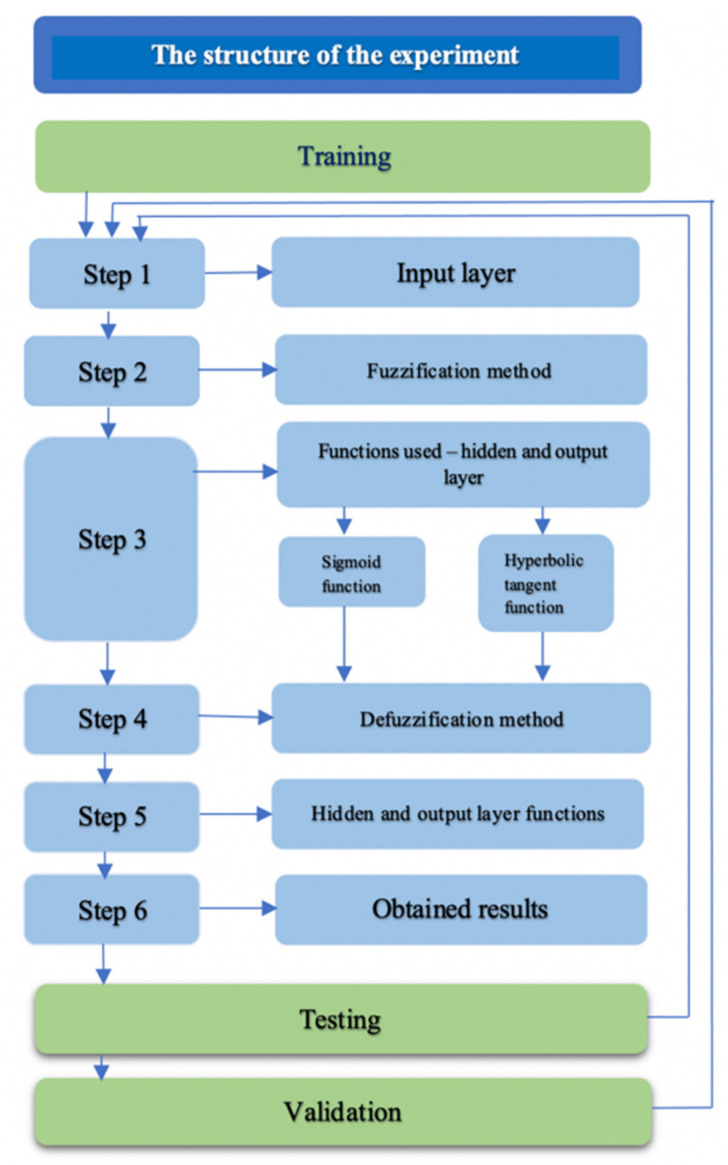
Algorithm for the robust design of the experiment.

The following robust design algorithm shows all three phases of our experiment with the selected methodology, which gave the best results, as shown in Figure 3.

The first phase of data training in the proposed experiment is described in the following steps:

**Step 1**. Input layer

The three input signals for the two proposed architectures ANN-L27 and ANN-L36 are: *X1* = E, *X2* = PEM_i_, and *X3* = KLOC, according to Formulas (1)–(7):

(1) $$Effort=A\times {\left[SIZE\right]}^{E}\times \overset{17}{\underset{i=1}{\prod }}EM_{i}$$

(2) $$E=B+0.01\times \overset{5}{\underset{j=1}{\sum }}SFj,\;where\;A=2.94,\;B=0.91$$

(3) $$Effort\left[PM\right]=2.94\times {\left[SIZE\right]}^{E}\times PEM_{i}$$

(4) $$PEM_{i}=\overset{17}{\underset{i=1}{\prod }}EM_{i}$$

(5) $$Time=C\times {\left(Effort\right)}^{F}$$

(6) $$F=D+0.2\times 0.01\times \overset{5}{\underset{j=1}{\sum }}SFj,\;where\;C=3.67,\;D=0.28$$

People = Effort/Time(7)

*A* and *B* are the fundamental constants for calibration; KSLOC (thousands of source lines of code) stated for the size of the software project; *SFj* represents five scale factors (prec, flex, resl, team, pmat); *EM_i_* stated for seventeen effort multipliers (rely, cplx, data, ruse, time, stor, pvol, acap, pcap, pcon, apex, plex, ltex, tool, sced, site, docu). In the input layer for the first phase of the experiment, 87 projects from the COCOMO 2000 dataset were used. They are divided into four clusters according to the actual effort value, as follows: small cluster (projects less than 90 PM), medium cluster (projects between 90 PM and 500 PM), large cluster (projects between 500 PM and 1000 PM), and very large cluster (projects larger than 1000 PM).

**Step 2.** Fuzzification method

In addition to the method of clustering, the different nature of the data needs to be further homogenized. This will be achieved by using the method of fuzzification [48,49], which involves mapping all three inputs. E, PEMi, and KLOC, into real values from the interval [0,1]. Function $\mu_{D}$(X):R→[0,1], μD=Xi−XminXmax−Xmin. *D* is the set of input data.

**Step 3.** The hidden and output layer functions use two different activation functions for both proposed ANN architectures. *EstEffANN* represents the output values of the proposed model.

*1.*  *Sigmoid function:*$y_{i=\frac{1}{1+e^{-x_{i}}},i=\bar{1,n}}$

Hidden and output layer functions for ANN-L27 architecture, according to Formulas (8)–(11):

(8) $$Y_{1}=\frac{1}{1+e^{-\left(X_{1}\cdot W_{1}+X_{2}\cdot W_{4}+X_{3}\cdot W_{7}\right)}}$$

(9) $$Y_{2}=\frac{1}{1+e^{-\left(X_{1}\cdot W_{2}+X_{2}\cdot W_{5}+X_{3}\cdot W_{8}\right)}}$$

(10) $$Y_{3}=\frac{1}{1+e^{-\left(X_{1}\cdot W_{3}+X_{2}\cdot W_{6}+X_{3}\cdot W_{9}\right)}}$$

(11) $$EstEffANN-L27=\frac{1}{1+e^{-\left(Y_{1}\cdot W_{10}+Y_{2}\cdot W_{11}+Y_{3}\cdot W_{12}+1\cdot W_{13}\right)}}$$

where *Y*_1_, *Y*_2_, *Y*_3_ are calculated values from the hidden layer and EstEffANN-L27 is the output value.

Hidden and output layer functions for ANN-L36 architecture, according to Formulas (12)–(18):

(12) $$Y_{1}=\frac{1}{1+e^{-\left(X_{1}\cdot W_{1}+X_{2}\cdot W_{5}+X_{3}\cdot W_{9}\right)}}$$

(13) $$Y_{2}=\frac{1}{1+e^{-\left(X_{1}\cdot W_{2}+X_{2}\cdot W_{6}+X_{3}\cdot W_{10}\right)}}$$

(14) $$\;Y_{3}=\frac{1}{1+e^{-\left(X_{1}\cdot W_{3}+X_{2}\cdot W_{7}+X_{3}\cdot W_{11}\right)}}$$

(15) $$Y_{4}=\frac{1}{1+e^{-\left(X_{1}\cdot W_{4}+X_{2}\cdot W_{8}+X_{3}\cdot W_{12}\right)}}$$

(16) $$Z_{1}=\frac{1}{1+e^{-\left(Y_{1}\cdot W_{13}+Y_{2}\cdot W_{15}+Y_{3}\cdot W_{17}+Y_{4}\cdot W_{19}\right)}}$$

(17) $$Z_{2}=\frac{1}{1+e^{-\left(Y_{1}\cdot W_{14}+Y_{2}\cdot W_{16}+Y_{3}\cdot W_{18}+Y_{4}\cdot W_{20}\right)}}$$

(18) $$EstEffANN-L36=\frac{1}{1+e^{-\left(Z_{1}\cdot W_{21}+Z_{2}\cdot W_{22}+1\cdot W_{23}\right)}}$$

*where Y_1_, Y_2_, Y_3_ and Y_4_ are calculated values from the first hidden layer, Z_1_ and Z_2_ are calculated values from the second hidden layer and EstEffANN-L36 is the output value.*

*2.*  *Hyperbolic tangent:* $y_{i=\frac{e^{x_{i}}-e^{-x_{i}}}{e^{-x_{i}}+e^{-x_{i}}},i=\bar{1,n}}$

Hidden and output layer functions for ANN-L27 architecture, according to Formulas (19)–(22):

(19) $$Y_{1}=\frac{e^{\left(X_{1}\cdot W_{1}+X_{2}\cdot W_{4}+X_{3}\cdot W_{7}\right)}-e^{-\left(X_{1}\cdot W_{1}+X_{2}\cdot W_{4}+X_{3}\cdot W_{7}\right)}}{e^{\left(X_{1}\cdot W_{1}+X_{2}\cdot W_{4}+X_{3}\cdot W_{7}\right)}+e^{-\left(X_{1}\cdot W_{1}+X_{2}\cdot W_{4}+X_{3}\cdot W_{7}\right)}}$$

(20) $$Y_{2}=\frac{e^{\left(X_{1}\cdot W_{2}+X_{2}\cdot W_{5}+X_{3}\cdot W_{8}\right)}-e^{-\left(X_{1}\cdot W_{2}+X_{2}\cdot W_{5}+X_{3}\cdot W_{8}\right)}}{e^{\left(X_{1}\cdot W_{2}+X_{2}\cdot W_{5}+X_{3}\cdot W_{8}\right)}+e^{-\left(X_{1}\cdot W_{2}+X_{2}\cdot W_{5}+X_{3}\cdot W_{8}\right)}}$$

(21) $$Y_{3}=\frac{e^{\left(X_{1}\cdot W_{3}+X_{2}\cdot W_{5}+X_{3}\cdot W_{9}\right)}-e^{-\left(X_{1}\cdot W_{3}+X_{2}\cdot W_{5}+X_{3}\cdot W_{9}\right)}}{e^{\left(X_{1}\cdot W_{3}+X_{2}\cdot W_{5}+X_{3}\cdot W_{9}\right)}+e^{-\left(X_{1}\cdot W_{3}+X_{2}\cdot W_{5}+X_{3}\cdot W_{9}\right)}}$$

(22) $$EstEffANN-L27=\frac{e^{\left(Y_{1}\cdot W_{10}+Y_{2}\cdot W_{11}+Y_{3}\cdot W_{12}+1\cdot W_{13}\right)}-e^{-\left(Y_{1}\cdot W_{10}+Y_{2}\cdot W_{11}+Y_{3}\cdot W_{12}+1\cdot W_{13}\right)}}{e^{\left(Y_{1}\cdot W_{10}+Y_{2}\cdot W_{11}+Y_{3}\cdot W_{12}+1\cdot W_{13}\right)}+e^{-\left(Y_{1}\cdot W_{10}+Y_{2}\cdot W_{11}+Y_{3}\cdot W_{12}+1\cdot W_{13}\right)}}$$

where *Y*_1_, *Y*_2_, *Y*_3_ are calculated values from the hidden layer and *EstEffANN-L27* is the output value.

Hidden and output layer functions for ANN-L36 architecture, according to Formulas (23)–(29):

(23) $$Y_{1}=\frac{e^{\left(X_{1}\cdot W_{1}+X_{2}\cdot W_{5}+X_{3}\cdot W_{9}\right)}-e^{-\left(X_{1}\cdot W_{1}+X_{2}\cdot W_{5}+X_{3}\cdot W_{9}\right)}}{e^{\left(X_{1}\cdot W_{1}+X_{2}\cdot W_{5}+X_{3}\cdot W_{9}\right)}+e^{-\left(X_{1}\cdot W_{1}+X_{2}\cdot W_{5}+X_{3}\cdot W_{9}\right)}}$$

(24) $$Y_{2}=\frac{e^{\left(X_{1}\cdot W_{2}+X_{2}\cdot W_{6}+X_{3}\cdot W_{10}\right)}-e^{-\left(X_{1}\cdot W_{2}+X_{2}\cdot W_{6}+X_{3}\cdot W_{10}\right)}}{e^{\left(X_{1}\cdot W_{2}+X_{2}\cdot W_{6}+X_{3}\cdot W_{10}\right)}+e^{-\left(X_{1}\cdot W_{2}+X_{2}\cdot W_{6}+X_{3}\cdot W_{10}\right)}}$$

(25) $$Y_{3}=\frac{e^{\left(X_{1}\cdot W_{3}+X_{2}\cdot W_{7}+X_{3}\cdot W_{11}\right)}-e^{-\left(X_{1}\cdot W_{3}+X_{2}\cdot W_{7}+X_{3}\cdot W_{11}\right)}}{e^{\left(X_{1}\cdot W_{3}+X_{2}\cdot W_{7}+X_{3}\cdot W_{11}\right)}+e^{-\left(X_{1}\cdot W_{3}+X_{2}\cdot W_{7}+X_{3}\cdot W_{11}\right)}}$$

(26) $$Y_{4}=\frac{e^{\left(X_{1}\cdot W_{4}+X_{2}\cdot W_{8}+X_{3}\cdot W_{12}\right)}-e^{-\left(X_{1}\cdot W_{4}+X_{2}\cdot W_{8}+X_{3}\cdot W_{12}\right)}}{e^{\left(X_{1}\cdot W_{4}+X_{2}\cdot W_{8}+X_{3}\cdot W_{12}\right)}+e^{-\left(X_{1}\cdot W_{4}+X_{2}\cdot W_{8}+X_{3}\cdot W_{12}\right)}}$$

(27) $$Z_{1}=\frac{e^{\left(Y_{1}\cdot W_{13}+Y_{2}\cdot W_{15}+Y_{3}\cdot W_{17}+Y_{4}\cdot W_{19}\right)}-e^{-\left(Y_{1}\cdot W_{13}+Y_{2}\cdot W_{15}+Y_{3}\cdot W_{17}+Y_{4}\cdot W_{19}\right)}}{e^{\left(Y_{1}\cdot W_{13}+Y_{2}\cdot W_{15}+Y_{3}\cdot W_{17}+Y_{4}\cdot W_{19}\right)}+e^{-\left(Y_{1}\cdot W_{13}+Y_{2}\cdot W_{15}+Y_{3}\cdot W_{17}+Y_{4}\cdot W_{19}\right)}}$$

(28) $$Z_{2}=\frac{e^{\left(Y_{1}\cdot W_{14}+Y_{2}\cdot W_{16}+Y_{3}\cdot W_{18}+Y_{4}\cdot W_{20}\right)}-e^{-\left(Y_{1}\cdot W_{14}+Y_{2}\cdot W_{16}+Y_{3}\cdot W_{18}+Y_{4}\cdot W_{20}\right)}}{e^{\left(Y_{1}\cdot W_{14}+Y_{2}\cdot W_{16}+Y_{3}\cdot W_{18}+Y_{4}\cdot W_{20}\right)}+e^{-\left(Y_{1}\cdot W_{14}+Y_{2}\cdot W_{16}+Y_{3}\cdot W_{18}+Y_{4}\cdot W_{20}\right)}}$$

(29) $$EstEffANN-L36=\frac{e^{\left(Z_{1}\cdot W_{21}+Z_{2}\cdot W_{22}+Y_{3}\cdot W_{18}+1\cdot W_{23}\right)}-e^{-\left(Z_{1}\cdot W_{21}+Z_{2}\cdot W_{22}+Y_{3}\cdot W_{18}+1\cdot W_{23}\right)}}{e^{\left(Z_{1}\cdot W_{21}+Z_{2}\cdot W_{22}+Y_{3}\cdot W_{18}+1\cdot W_{23}\right)}+e^{-\left(Z_{1}\cdot W_{21}+Z_{2}\cdot W_{22}+Y_{3}\cdot W_{18}+1\cdot W_{23}\right)}}$$

where *Y*_1_, *Y*_2_, *Y*_3_ and *Y*_4_ are calculated values from the first hidden layer, *Z*_1_ and *Z*_2_ are calculated values from the second hidden layer and *EstEffANN-L36* is the output value. 

Weight factors in both proposed architectures take initial values from the interval [−1, 0, 1] depending on L1, L2, and L3 levels. After performing the first iteration according to the Taguchi orthogonal vector plan, the value of the cost effect function is calculated for each of the listed architectures based on Table 1 and Table 2. Calculating the levels for ANN-L27 architecture [1,3] according to Formula (30):

L1W_1_ = cost1 + cost2 + ⋯ + cost9  
L2W_1_ = cost10 + cost11 + ⋯ + cost18 L3W_1_ = cost19 + cost20 + ⋯ + cost27 …               L1W_13_ = cost1 + cost5 + ⋯ + cost26 L1W_13_ = cost2 + cost6 + ⋯ + cost27 L1W_13_ = cost3 + cost4 + ⋯ + cost25 where cost(***i***) = Σ MRE(ANN-L27(***i***)) (30)

Calculating the levels for ANN-L36 architecture [1,3] according to Formula (31):

L1W_1_ = cost1 + cost2 + ⋯ + cost18  L2W_1_ = cost19 + cost20 + ⋯ + cost36 …               L1W_23_ = cost1 + cost5 + ⋯ + cost34 L2W_23_ = cost2 + cost6 + ⋯ + cost35 L3W_23_ = cost3 + cost4 + ⋯ + cost36 where cost(***i***) = Σ MRE(ANN-L36(***i***)) (31)

For each subsequent iteration, the interval is divided as follows [1], according to Formula (32):

L1W_1_new = L2W_1_old L2W_1_new = L2W_1_old + (L3W1old − L2W1old)/2 L3W_1_new = L3W_1_old (32)

where the suffix “old” means values from the interval of the previous iteration, and “new” means the value calculated based on the division of the previous intervals.

**Step 4.** Defuzzification method

Defuzzification of the data in all three phases of the experiment on four datasets is performed as follows, according to Formulas (33)–(35):

(33) $$X_{i}=(X_{min}+\mu_{D}\left(X_{i}\right))\cdot (X_{max}-X_{min})$$

(34) $$\mathrm{OA}(\mathrm{ANN}\text{-}L27)=X_{i},\;i=\bar{\left(1,\;27\right)}$$

(35) $$\mathrm{OA}(\mathrm{ANN}\text{-}L36)=X_{i},\;i=(\bar{1,\;36)}$$

**Step 5.** Hidden and output layer

The values of the output layer were calculated using the following Formulas (36)–(38), [1]:

(36) $$MAE_{i}=\frac{1}{n}\Sigma_{i=1}^{n}|\mathrm{ActEffort}\;\mathrm{EstEffort}|\;$$

*MRE* = Deviation/Actual_Effort (37)

(38) $$MRE=\frac{1}{n}\sum_{i=1}^{n}MRE_{i},\;\mathrm{MMRE}=\mathrm{mean}(\mathrm{MRE})\;$$

In the first step, the deviation as the difference between the actual and estimated value is calculated. Then, the mean absolute error (MAE) and MRE values for each ANN (i) network for both proposed architectures, as well as the MMRE of each iteration performed, were calculated. Finally, the gradient descent (GA) for both ANN architectures is calculated in all three phases of the experiment. The GA value in our experiment is 0.01 (GA < 0.01) and is present as follows, according to Formula (39):

(39) $$GA=MRE_{i1}-MRE_{i2}\;<\;0.01,\;\mathrm{where}\;I=\bar{(1,\;n}),\;n\;\mathrm{is}\;a\;\mathrm{number}\;\mathrm{of}\;\mathrm{ANN}\;$$

By examining correlation coefficients, such as Spearman’s rho and Pearson’s coefficient [50,51], we will determine whether the agreement between the actual and estimated value is good, i.e., whether our approach is reliable and efficient, using Formulas (40)–(43):

(40) $$PRED\left(x\right)=\frac{1}{n}\overset{n}{\underset{i=1}{\sum }}\left\{\begin{array}{c} 1,\;if\;MRE\leq x\\ 0,\;otherwise \end{array}\right.$$

PRED(k) = count(MRE) < 25%(41)

PRED(k) = count(MRE) < 30%(42)

PRED(k) = count(MRE) < 50%, where k = 25, 30 and 50, respectively(43)

During all three phases of the experiment, the prediction was monitored as another important indicator of the reliability and safety of our proposed approach.

**Step 6.** The obtained results are presented and discussed in the next section.

The second phase of our experiment is data testing, which is performed according to the same robust design algorithm on the same dataset but with other selected projects. The third phase of our experiment is validation, which is also performed by the same algorithm but on different datasets. Finally, testing and validation are performed on the Winner network (the network that gives the best results for each of the two proposed ANN architectures).

## 4. Obtained Results

In the first phase of training using the first proposed activation sigmoid function, the number of iterations on a small cluster was 6, while in the medium cluster it was 5. For a large cluster and a very large cluster, the number of required iterations was 7. The lowest MMRE value is achieved with the proposed ANN-L27 architecture over a large cluster (38.6%). Both ANNs achieve similar MMRE values over the medium cluster; for ANN-L27, the MMRE value is 40.9%, while for ANN-L36, the MMRE value is 40.1%. The worst result is achieved with a small cluster; for ANN-L27, the value of MMRE is 58.0%, while for ANN-L36, this value is 55.9%. The mean MMRE on the entire dataset (Dataset_1) in the training phase is better for the ANN-L27 network by 1% and is 48.2%, shown in Table 5.

In the second phase of testing, using the first proposed activation sigmoid function, the number of iterations is the same as in the previous phase. The best result of the MMRE value is achieved by a large cluster with ANN-L36 architecture (16.1%), while the worst result is achieved with a small cluster of both proposed architectures. At this stage of the experiment, the mean value of MRE over (Dataset_2) is better in ANN-L36 by 2.3% and is 37.4% Table 5.

The third phase of the experiment includes four validation datasets of the first proposed activation sigmoid function. In the first validation set, the best result is achieved with a small cluster of the proposed ANN-L27 architecture (37.5%), while the worst value is achieved on the medium cluster of the same architecture and is 57.8%. The mean value of MMRE over (Dataset_3) on all clusters is 8.5% better in the proposed ANN-L36 architecture and is 38.8%. In the second validation set, the lowest value of MMRE is achieved on a large cluster with ANN-L36 architecture (17.6%), while the worst result is achieved on a small cluster of ANN-L27 architecture (68.4%). The mean MMRE value over (Dataset_4) is 3.9% better in the proposed ANN-L36 architecture than in ANN-L27 and is 40.0%. In the third validation set, the best result was achieved by the ANN-L27 architecture on a large cluster (20.6%). The mean value of MMRE over (Dataset_5) is better by 1.5% with the ANN-L27 architecture and is 35.1%. In the fourth validation set, the best result is achieved by a small cluster with ANN-L36 architecture (15.9%), and the total observed mean value of MMRE over (Dataset_6) is better with the proposed architecture ANN-L27 by 1% and is 30.1%, as shown in Table 5.

**Figure 4:**
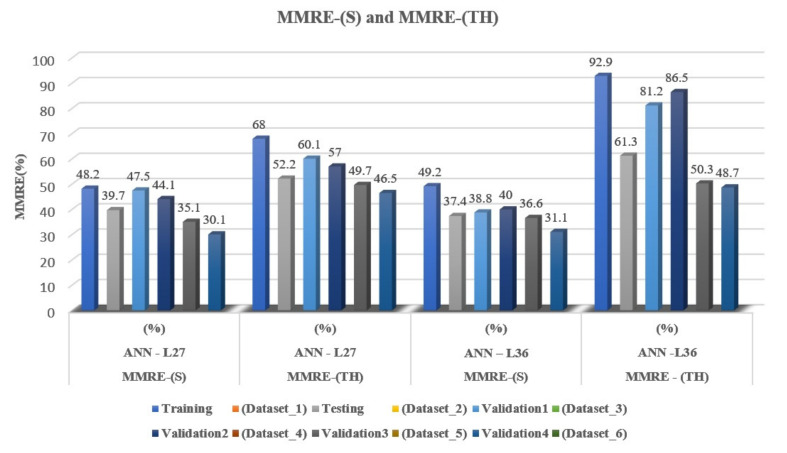
Graphical representation comparing MMRE values with two different activation functions.

Analogous to the previous phases of the experiment, in all calculations, instead of the sigmoid activation function, a hyperbolic tangent activation function was used. By comparative analysis of the obtained results, using two different activation functions on two proposed architectures and six different datasets, it can be concluded that the hyperbolic tangent function as activation gives 1.5–2-times worse results of MMRE values compared to the sigmoid activation function. In the first training phase, the MMRE value was significantly better for the ANN-L27 architecture and was 68.0%. In the second phase of testing, the ANN-L27 architecture gives a better MMRE value (52.2%). In all experimental validation sets, the ANN-L27 architecture gives better results, although they are much worse than the first proposed sigmoid activation function (Table 6, Figure 4).

The correlation value between actual effort and estimated effort was monitored during all phases of the experiment on all datasets for both proposed architectures. It can be concluded that both correlation coefficients in the first approach using the sigmoid activation function are greater than 0.5, and that the highest correlation coefficient on Dataset_4 and according to Spearman is 0.997 for the ANN-L27 network and 0.994 for the ANN-L36 network. In the second approach, where the hyperbolic tangent activation function was used, the values of the correlation coefficients were not calculated because the MMRE value was unsatisfactory, as shown in Table 7.

As another confirmation of our proposed experiment’s durability, robustness, and accuracy, the prediction was monitored during all three phases on the six datasets used for the proposed architectures in the first approach of the sigmoid activation function. The prediction on three different criteria, 25, 30, and 50, showed that a large number of projects meet the given conditions. The prediction on the 25% criterion is the best in the second phase of testing, and is 50% for ANN-L36. The prediction on the 30% criterion achieves the best result in the third validation set of 56.3% for ANN-L36. Finally, the 50% criterion prediction is 100% in both the testing phase for the ANN-L27 architecture and the third validation set for the ANN-L36, as shown in Table 8.

**Figure 5:**
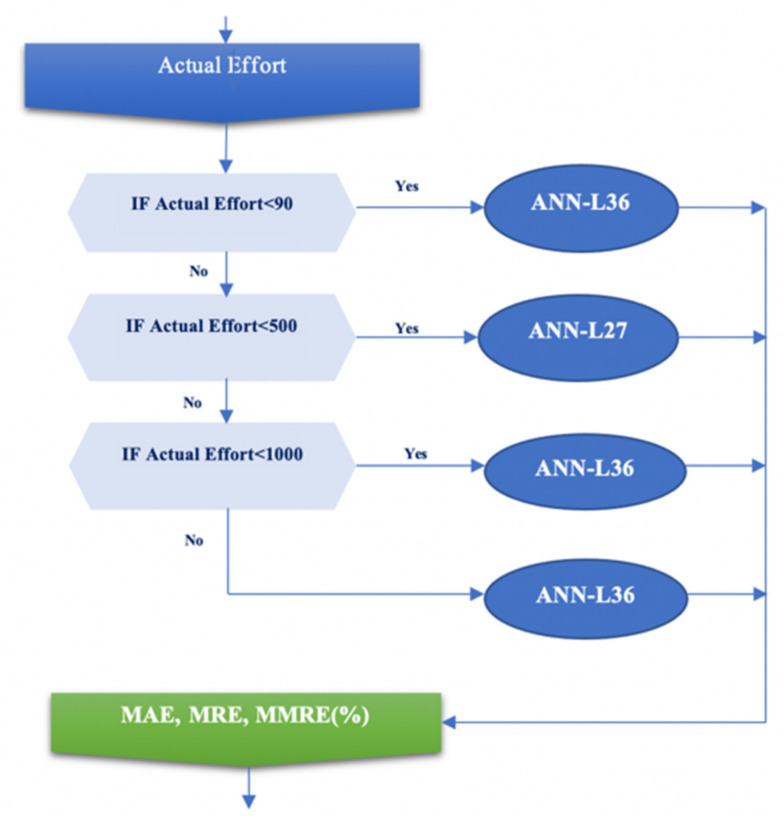
Selection of the appropriate ANN architecture for estimating the MMRE value of a particular scenario.

The main goal of our experiment is to find the ANN architecture that gives the best value for MMRE. In this experiment, a more complex ANN-L36 architecture was chosen, which, unlike the ANN-L27, has two hidden layers, in the expectation that it will give a better MMRE value. Upon completing our experiment, we found that the new, more complex ANN-L36 architecture yields a 1% better error value than the proposed ANN-L27. This leads to the conclusion that a more complex architecture does not give a significantly better error value. On the other hand, for the needs of the software industry and the creation of an adequate tool that will be able to estimate the obtained parameter successfully, the obtained error value can be used depending on the size of the project on specific clusters, as shown in Figure 5.

### Support Vector Regression in Machine Learning

In order to confirm the correctness and reliability of the proposed approach and its comparison with other artificial intelligence tools, the Support Vector Regression (SVR) algorithm was used. SVR is a popular machine-learning (ML) algorithm and stems from the use of observed data for training. The SVR is a robust and proficient technique for both classification and regression. In addition, it minimizes the expected error, thus reducing the problem of overfitting [52,53]. The SVR machine algorithm divides the plane by the function f into two parts, so that the points (project input values) lie above or below the function f. Three different functions inside a kernel in SVR with radial basis function (RBF) were used:

- linear kernel function,
- quadratic kernel function,
- cubic kernel function.

**Figure 6:**
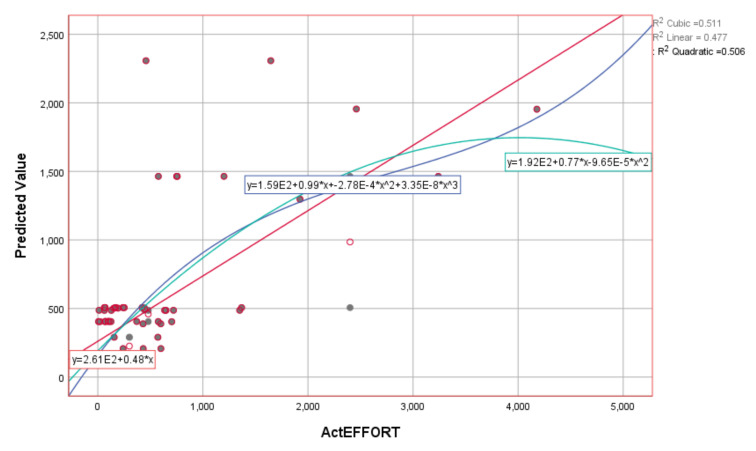
Graphical representation using different kernel functions based on SVR (RBF) for ActEffort on the training dataset.

**Figure 7:**
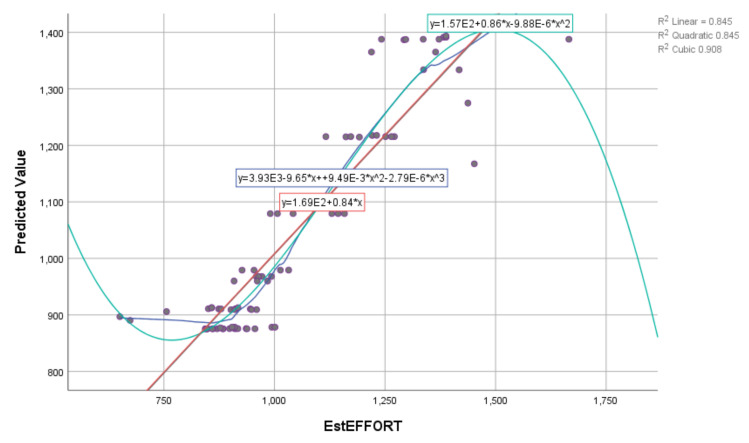
Graphical representation using different kernel functions based on SVR (RBF) for EstEffort on the training dataset.

The obtained results show that the estimated value in the training part of the experiment, based on three input variables of E, PEM_i_, and KLOC, has a very high degree of correlation (deterministic coefficient—R^2^). The R^2^ values are shown in Table 9. Graphical representations of the actual and estimated values using the SVR algorithm and the corresponding kernel functions are shown in Figure 6 and Figure 7.

The MMRE value for all three kernel functions used in the SVR algorithm is slightly higher than the proposed approach using ANN and Taguchi orthogonal arrays. Based on the obtained results, it can be concluded that our proposed approach is stable, reliable, and efficient even when used by other machine-learning tools.

## 5. Threats to Validity

### 5.1. Internal Validity

The choice of methods, appropriate coding and decoding techniques, and the value assignment for all the experimental parameters in the presented study are common threats to internal validity in the literature on model-based software effort estimation. In our experiment, all these validity threats were already handled using hyperparameter optimization via the Taguchi method, based on orthogonal arrays (a unique set of Latin squares), which demonstrated an effective apparatus in a robust design, i.e., an optimization search technique. This robust design method combines the input values (signals) with weights using the appropriate activation function to obtain the output value. One of the requirements in this experiment is to obtain the smallest number of iterations performed to shorten the estimation time. Two simple architectures, constructed according to appropriate orthogonal plans to avoid overfitting, are presented. The presented experiment shows the efficient application and the required accuracy of estimating the convergence rate of the proposed architectures. Our approach uses the orthogonal array tuning method (OATM), which always gives good results and requires a much smaller number of experiments to find the optimal solution. Using the OATM method, the hyperparameters are the levels. In our proposed model, we use three levels: L1, L2, and L3 according to works [1,2,3], and, based on these levels, we build the F-L (factor-level) table. The optimum parameter set is believed to be the most suitable setup for each test project case in the experiments.

### 5.2. External Validity

This study used six well-known datasets with sufficient project data metrics (observations greater than an unknown number of factors to be determined). In addition, two proposed ANN architectures with 13 and 23 parameters for ANN-L27 and ANN-L36, respectively, were used. The COCOMO attributes were used as predictors of scale factors, cost drivers, and software size in KLOC. These projects are collected from different companies and countries, and they are heterogeneous in terms of their features: this makes them challenging for evaluating the effort estimation with ANN architectures in software development project techniques. By dividing the interval in half at every iteration, the search converges extremely quickly, i.e., in fewer than 10 iterations (the search interval for a weight shrinks three orders of magnitude in ten steps). The orthogonal array tuning method (OATM) proposed always gives good results and requires a much smaller number of experiments, i.e., observations (historical project data) in the dataset, to find the optimal solution. However, we believe that it will benefit from replicating this study using other software project datasets.

## 6. Conclusions and Future Work

In the presented experiment, we showed that the estimation of software projects when minimizing MMRE does not depend exclusively on the complexity of the ANN architecture. We found that the error in the ANN-L36 architecture was 1% better than the error in the ANN-L27 architecture. In our approach, using clustering and fuzzification methods, we have shown that the proposed models are reliable, efficient, accurate, and applicable to many projects. We conclude that there is a big difference in the choice of activation functions, where, when using the sigmoid activation function concerning the hyperbolic tangent, we get 1.5 to 2-times better results. Therefore, correlation and prediction parameters for models that used the hyperbolic tangent activation function were not monitored in further analyses. The main advantages of our proposed approach are that the number of iterations is small (fewer than 8), which significantly shortens the estimation time, the simple ANN architecture of the two proposed networks ANN-L27 and ANN-L36, the high coverage of different values of software projects which was checked on six different datasets, and minimum MMRE value. Possible disadvantages of the proposed approach may be further reductions in MMRE knowledge using other methods and datasets, including the size of attributes expressed in source code lines. However, there are no particular restrictions to the application of this approach. The obtained results can be used to construct an appropriate tool that would allow software companies, software engineers, project managers, and test engineers to efficiently, quickly, and safely assess the project’s development using the fundamental value of actual effort in a particular cluster. Future research is focused on constructing a tool based on the idea proposed in this paper. In addition, the use of a different number of input signals and checking over other attribute sizes is in progress. Advantages and limitations of the proposed approach are:

- The choice of methods, data normalization techniques, and the value assigned for all the experimental parameters are common limitations in the literature on model-based software effort estimation. In the presented study, it is, however, our belief that all these limitations were already handled by the use of hyperparameter optimization via the Taguchi method based on orthogonal arrays (a unique set of Latin squares);
- The presented experiment in the paper has been tested and validated several times through different data sets, thus achieving an extensive coverage of input sizes (project values);
- The minimum number of iterations was performed, i.e., shortened required execution time;
- With this approach, we achieved a stable and reliable estimation accuracy that is acceptable compared to other estimation models and software development efforts, based on three attributes as in the COCOMO estimation model;
- According to ANN architecture, and constructions based on orthogonal vector plans, other estimation techniques, such as use case point and functional point, will be considered.

## Figures and Tables

**Table 1:** Taguchi orthogonal array OA(3^13^) for ANN-L27.

ANN-L27	W_1_	W_2_	W_3_	W_4_	W_5_	W_6_	W_7_	W_8_	W_9_	W_10_	W_11_	W_12_	W_13_
ANN1	L1	L1	L1	L1	L1	L1	L1	L1	L1	L1	L1	L1	L1
ANN2	L1	L1	L1	L1	L2	L2	L2	L2	L2	L2	L2	L2	L2
ANN3	L1	L1	L1	L1	L3	L3	L3	L3	L3	L3	L3	L3	L3
ANN4	L1	L2	L2	L2	L1	L1	L1	L2	L2	L2	L3	L3	L3
ANN5	L1	L2	L2	L2	L2	L2	L2	L3	L3	L3	L1	L1	L1
ANN6	L1	L2	L2	L2	L1	L1	L1	L3	L3	L3	L2	L2	L2
ANN7	L1	L3	L3	L3	L1	L1	L1	L3	L3	L3	L2	L2	L2
ANN8	L1	L3	L3	L3	L2	L2	L2	L1	L1	L1	L3	L3	L3
ANN9	L1	L3	L3	L3	L3	L3	L3	L2	L2	L2	L1	L1	L1
ANN10	L2	L1	L2	L3	L1	L2	L3	L1	L2	L3	L1	L2	L3
ANN11	L2	L1	L2	L3	L2	L3	L1	L2	L3	L1	L2	L3	L1
ANN12	L2	L1	L2	L3	L3	L1	L2	L3	L1	L2	L3	L1	L2
ANN13	L2	L2	L3	L1	L1	L2	L3	L2	L3	L1	L3	L1	L2
ANN14	L2	L2	L3	L1	L2	L3	L1	L3	L1	L2	L1	L2	L3
ANN15	L2	L2	L3	L1	L3	L1	L2	L1	L2	L3	L2	L3	L1
ANN16	L2	L3	L1	L2	L1	L2	L3	L3	L1	L2	L2	L3	L1
ANN17	L2	L3	L1	L2	L2	L3	L1	L1	L2	L3	L3	L1	L2
ANN18	L2	L3	L1	L2	L3	L1	L2	L2	L3	L1	L1	L2	L3
ANN19	L3	L1	L3	L2	L1	L3	L2	L1	L3	L2	L1	L3	L2
ANN20	L3	L1	L3	L2	L2	L1	L3	L2	L1	L3	L2	L1	L3
ANN21	L3	L1	L3	L2	L3	L2	L1	L3	L2	L1	L3	L2	L1
ANN22	L3	L2	L1	L3	L1	L3	L2	L2	L1	L3	L3	L2	L1
ANN23	L3	L2	L1	L3	L2	L1	L3	L3	L2	L1	L1	L3	L2
ANN24	L3	L2	L1	L3	L3	L2	L1	L1	L3	L2	L2	L1	L3
ANN25	L3	L3	L2	L1	L1	L3	L2	L3	L2	L1	L2	L1	L3
ANN26	L3	L3	L2	L1	L2	L1	L3	L1	L3	L2	L3	L2	L1
ANN27	L3	L3	L2	L1	L3	L2	L1	L2	L1	L3	L1	L3	L2

**Table 2:** Taguchi orthogonal array OA(2^11^3^12^) for ANN-L36.

ANN-L36	W_1_	W_2_	W_3_	W_4_	W_5_	W_6_	W_7_	W_8_	W_9_	W_10_	W_11_	W_12_	W_13_	W_14_	W_15_	W_16_	W_17_	W_18_	W_19_	W_20_	W_21_	W_22_	W_23_
ANN1	L1	L1	L1	L1	L1	L1	L1	L1	L1	L1	L1	L1	L1	L1	L1	L1	L1	L1	L1	L1	L1	L1	L1
ANN2	L1	L1	L1	L1	L1	L1	L1	L1	L1	L1	L1	L2	L2	L2	L2	L2	L2	L2	L2	L2	L2	L2	L2
ANN3	L1	L1	L1	L1	L1	L1	L1	L1	L1	L1	L1	L3	L3	L3	L3	L3	L3	L3	L3	L3	L3	L3	L3
ANN4	L1	L1	L1	L1	L1	L2	L2	L2	L2	L2	L2	L1	L1	L1	L1	L2	L2	L2	L2	L3	L3	L3	L3
ANN5	L1	L1	L1	L1	L1	L2	L2	L2	L2	L2	L2	L2	L2	L2	L2	L3	L3	L3	L3	L1	L1	L1	L1
ANN6	L1	L1	L1	L1	L1	L2	L2	L2	L2	L2	L2	L3	L3	L3	L3	L1	L1	L1	L1	L2	L2	L2	L2
ANN7	L1	L1	L2	L2	L2	L1	L1	L1	L2	L2	L2	L1	L1	L2	L3	L1	L2	L3	L3	L1	L2	L2	L3
ANN8	L1	L1	L2	L2	L2	L1	L1	L1	L2	L2	L2	L2	L2	L3	L1	L2	L3	L1	L1	L2	L3	L3	L1
ANN9	L1	L1	L2	L2	L2	L1	L1	L1	L2	L2	L2	L3	L3	L1	L2	L3	L1	L2	L2	L3	L1	L1	L2
ANN10	L1	L2	L1	L2	L2	L1	L2	L2	L1	L1	L2	L1	L1	L3	L2	L1	L3	L2	L3	L2	L1	L3	L2
ANN11	L1	L2	L1	L2	L2	L1	L2	L2	L1	L1	L2	L2	L2	L1	L3	L2	L1	L3	L1	L3	L2	L1	L3
ANN12	L1	L2	L1	L2	L2	L1	L2	L2	L1	L1	L2	L3	L3	L2	L1	L3	L2	L1	L2	L1	L3	L2	L1
ANN13	L1	L2	L2	L1	L2	L2	L1	L2	L1	L2	L1	L1	L2	L3	L1	L3	L2	L1	L3	L3	L2	L1	L2
ANN14	L1	L2	L2	L1	L2	L2	L1	L2	L1	L2	L1	L2	L3	L1	L2	L1	L3	L2	L1	L1	L3	L2	L3
ANN15	L1	L2	L2	L1	L2	L2	L1	L2	L1	L2	L1	L3	L1	L2	L3	L2	L1	L3	L2	L2	L1	L3	L1
ANN16	L1	L2	L2	L2	L1	L2	L2	L1	L2	L1	L1	L1	L2	L3	L2	L1	L1	L3	L2	L3	L3	L2	L1
ANN17	L1	L2	L2	L2	L1	L2	L2	L1	L2	L1	L1	L2	L3	L1	L3	L2	L2	L1	L3	L1	L1	L3	L2
ANN18	L1	L2	L2	L2	L1	L2	L2	L1	L2	L1	L1	L3	L1	L2	L1	L3	L3	L2	L1	L2	L2	L1	L3
ANN19	L2	L1	L2	L2	L1	L1	L2	L2	L1	L2	L1	L1	L2	L1	L3	L3	L3	L1	L2	L2	L1	L2	L3
ANN20	L2	L1	L2	L2	L1	L1	L2	L2	L1	L2	L1	L2	L3	L2	L1	L1	L1	L2	L3	L3	L2	L3	L1
ANN21	L2	L1	L2	L2	L1	L1	L2	L2	L1	L2	L1	L3	L1	L3	L2	L2	L2	L3	L1	L1	L3	L1	L2
ANN22	L2	L1	L2	L1	L2	L2	L2	L1	L1	L1	L2	L1	L2	L2	L3	L3	L1	L2	L1	L1	L3	L3	L2
ANN23	L2	L1	L2	L1	L2	L2	L2	L1	L1	L1	L2	L2	L3	L3	L1	L1	L2	L3	L2	L2	L1	L1	L3
ANN24	L2	L1	L2	L1	L2	L2	L2	L1	L1	L1	L2	L3	L1	L1	L2	L2	L3	L1	L3	L3	L2	L2	L1
ANN25	L2	L1	L1	L2	L2	L2	L1	L2	L2	L1	L1	L1	L3	L2	L1	L2	L3	L3	L1	L3	L1	L2	L2
ANN26	L2	L1	L1	L2	L2	L2	L1	L2	L2	L1	L1	L2	L1	L3	L2	L3	L1	L1	L2	L1	L2	L3	L3
ANN27	L2	L1	L1	L2	L2	L2	L1	L2	L2	L1	L1	L3	L2	L1	L3	L1	L2	L2	L3	L2	L3	L1	L1
ANN28	L2	L2	L2	L1	L1	L1	L1	L2	L2	L1	L2	L1	L3	L2	L2	L2	L1	L1	L3	L2	L3	L1	L3
ANN29	L2	L2	L2	L1	L1	L1	L1	L2	L2	L1	L2	L2	L1	L3	L3	L3	L2	L2	L1	L3	L1	L2	L1
ANN30	L2	L2	L2	L1	L1	L1	L1	L2	L2	L1	L2	L3	L2	L1	L1	L1	L3	L3	L2	L1	L2	L3	L2
ANN31	L2	L2	L1	L2	L1	L2	L1	L1	L1	L2	L2	L1	L3	L3	L3	L2	L3	L2	L2	L1	L2	L1	L1
ANN32	L2	L2	L1	L2	L1	L2	L1	L1	L1	L2	L2	L2	L1	L1	L1	L3	L1	L3	L3	L2	L3	L2	L2
ANN33	L2	L2	L1	L2	L1	L2	L1	L1	L1	L2	L2	L3	L2	L2	L2	L1	L2	L1	L1	L3	L1	L3	L3
ANN34	L2	L2	L1	L1	L2	L1	L2	L1	L2	L2	L1	L1	L3	L1	L2	L3	L2	L3	L1	L2	L2	L3	L1
ANN35	L2	L2	L1	L1	L2	L1	L2	L1	L2	L2	L1	L2	L1	L2	L3	L1	L3	L1	L2	L3	L3	L1	L2
ANN36	L2	L2	L1	L1	L2	L1	L2	L1	L2	L2	L1	L3	L2	L3	L1	L2	L1	L2	L3	L1	L1	L2	L3

**Table 3:** Information about datasets in all three phases of the experiment.

	Dataset	Number of Projects	Phase in Experiment
**Dataset_1**	COCOMO 2000 dataset	87	Training
**Dataset_2**	COCOMO 2000 dataset	23	Testing
**Dataset_3**	COCOMO 81	46	Validation1
**Dataset_4**	NASA dataset	60	Validation2
**Dataset_5**	Kemerer dataset + Industrial projects	15 + 5	Validation3
**Dataset_6**	Desharnais	80	Validation4

**Table 4:** The basic statistics regarding datasets in all three phases of the experiment.

Dataset	Number of Projects	Min [PM]	Max [PM]	Mean [PM]	Stand Deviation [PM]
**Dataset_1**	87	8.4	8211.0	630.9	1181.3
**Dataset_2**	23	12.0	1924.5	487.8	559.3
**Dataset_3**	46	33.0	6599.9	651.9	1373.9
**Dataset_4**	60	8.4	3240.0	406.4	656.9
**Dataset_5**	20	23.2	1780.0	408.6	462.1
**Dataset_6**	80	54.6	2394.0	504.6	441.8

**Table 5:** MMRE values for both ANNs in all three phases.

MMRE	Cluster	MMRE ANN-L27 (%)	Mean (MMRE) ANN-L27 (%)	MMRE ANN-L36 (%)	Mean (MMRE) ANN-L36 (%)
**Training** **(Dataset_1)**	**Small**	58.0	48.2	55.9	49.2
	**Medium**	40.9		40.1	
	**Large**	38.6		47.8	
	**Very Large**	55.4		52.9	
**Testing** **(Dataset_2)**	**Small**	54.9	39.7	57.4	37.4
	**Medium**	30.8		31.3	
	**Large**	20.3		16.1	
	**Very Large**	52.7		44.8	
**Validation 1** **(Dataset_3)**	**Small**	37.5	47.5	42.4	38.8
	**Medium**	57.8		34.8	
	**Large**	40.6		53.6	
	**Very Large**	47.2		39.2	
**Validation 2** **(Dataset_4)**	**Small**	68.4	44.1	59.9	40.0
	**Medium**	45.7		41.2	
	**Large**	19.2		17.6	
	**Very Large**	43.0		41.9	
**Validation 3** **(Dataset_5)**	**Small**	39.7	35.1	37.8	36.6
	**Medium**	41.6		46.0	
	**Large**	20.6		25.7	
	**Very Large**	38.2		36.9	
**Validation 4** **(Dataset_6)**	**Small**	16.9	30.1	15.9	31.1
	**Medium**	35.8		36.0	
	**Large**	29.0		34.4	
	**Very Large**	38.8		38.0	

**Table 6:** Comparison of MMRE values using sigmoid (S) and hyperbolic tangent (TH) activation function.

MMRE	MMRE-(S) ANN-L27 (%)	MMRE-(TH) ANN-L27 (%)	MMRE-(S) ANN-L36 (%)	MMRE-(TH) ANN-L36 (%)
**Training** **(Dataset_1)**	48.2	68.0	49.2	92.9
**Testing** **(Dataset_2)**	39.7	52.2	37.4	61.3
**Validation1** **(Dataset_3)**	47.5	60.1	38.8	81.2
**Validation2** **(Dataset_4)**	44.1	57.0	40.0	86.5
**Validation3** **(Dataset_5)**	35.1	49.7	36.6	50.3
**Validation4** **(Dataset_6)**	30.1	46.5	31.1	48.7

**Table 7:** Values of correlation coefficients.

Correlation ActEffort and EstEffort	Pearson ANN-L27	Pearson ANN-L36	Spearman’s rho ANN-L27	Spearman’s rho ANN-L36
**Dataset_1**	0.573	0.603	0.866	0.910
**Dataset_2**	0.937	0.962	0.873	0.874
**Dataset_3**	0.591	0.638	0.542	0.851
**Dataset_4**	0.836	0.765	0.997	0.944
**Dataset_5**	0.824	0.837	0.541	0.547
**Dataset_6**	0.779	0.773	0.857	0.782

**Table 8:** Prediction values for both architectures.

PRED (%)	PRED (25) ANN-L27	PRED (25) ANN-L36	PRED (30) ANN-L27	PRED (30) ANN-L36	PRED (50) ANN-L27	PRED (50) ANN-L36
**Training**	17.8	17.8	24.4	23.3	47.8	46.7
**Testing**	45.0	50.0	50.0	55.0	100.0	95.0
**Validation1**	27.5	32.5	32.5	42.5	62.5	72.5
**Validation2**	18.3	18.3	21.7	26.7	38.3	46.7
**Validation3**	25.0	31.3	45.0	56.3	90.0	100.0
**Validation4**	35.0	36.3	46.3	45.0	85.0	77.5

**Table 9:** R^2^ values using different kernel functions based on SVR (RBF).

SVR (RBF)	ActEffort	EstEffort	MMRE (%)
R^2^ Linear	0.477	0.845	52.9%
R^2^ Cubic	0.511	0.908	63.7%
R^2^ Quadratic	0.506	0.845	68.4%

## Data Availability

This study is based on the research and data that will be presented in detail in the doctoral dissertation of Nevena Rankovic.

## References

[1] Stoica A., Blosiu J. (1997). Neural Learning using orthogonal arrays. Adv. Intell. Syst..

[2] Khaw J.F.C., Lim B.S., Lim L.E.N. (1995). Optimal design of neural networks using the Taguchi method. Neurocomputing.

[3] Rankovic N., Rankovic D., Ivanovic M., Lazic L. (2021). A New Approach to Software Effort Estimation Using Different Artificial Neural Network Architectures and Taguchi Orthogonal Arrays. IEEE Access.

[4] Boehm B.W. Software cost estimation meets software diversity. Proceedings of the 39th International Conference on Software Engineering Companion (ICSE-C’17).

[5] Boehm B., Abts C., Chulani S. (2000). Software development cost estimation approaches-A survey. Ann. Softw. Eng..

[6] Fadhil A.A., Alsarraj R.G., Altaie A.M. (2020). Software Cost Estimation Based on Dolphin Algorithm. IEEE Access.

[7] Kumar P.S., Behera H.S., Kumari A., Nayak J., Naik B. (2020). Advancement from neural networks to deep learning in software effort estimation: Perspective of two decades. Comput. Sci. Rev..

[8] Saavedra Martínez J.I., Valdés Souto F., Rodríguez Monje M. Analysis of automated estimation models using machine learning. Proceedings of the 8th International Conference in Software Engineering Research and Innovation (CONISOFT).

[9] Mahmood Y., Kama N., Azmi A., Khan A.S., Ali M. (2021). Software Effort Estimation Accuracy Prediction of Machine Learning Techniques: A Systematic Performance Evaluation. J. Softw. Pract. Exp..

[10] BaniMustafa A. Predicting software effort estimation using machine learning techniques. Proceedings of the 8th International Conference on Computer Science and Information Technology (CSIT 2018).

[11] Langsari K., Sarno R. Optimizing effort and time parameters of COCOMO II estimation using fuzzy multiobjective PSO. Proceedings of the 4th International Conference on Electrical Engineering, Computer Science and Informatics (EECSI).

[12] Gharehchopogh F.S. Neural networks application in software cost estimation: A case study. Proceedings of the 2011 International Symposium on Innovations in Intelligent Systems and Applications.

[13] Kumar P.S., Behera H.S. (2020). Estimating Software Effort Using Neural Network: An Experimental Investigation. Computational Intelligence in Pattern Recognition.

[14] Boehm B.W., Abts C., Brown A.W., Chulani S., Clark B.K., Horowitz E. (2000). Software Cost Estimation with Cocomo II.

[15] Nassif A.B., Azzeh M., Idri A., Abran A. (2019). Software development effort estimation using regression fuzzy models. Comput. Intell. Neurosci..

[16] Safari S., Erfani A.R. (2020). A new method for fuzzification of nested dummy variables by fuzzy clustering membership functions and its application in financial economy. Iran. J. Fuzzy Syst..

[17] Kaushik A., Tayal D.K., Yadav K. (2020). A fuzzified story point approach for agile projects. Int. J. Agil. Syst. Manag..

[18] Orthogonal Arrays (Taguchi Designs). https://www.york.ac.uk/depts/maths/tables/orthogonal.htm.

[19] Taguchi Orthogonal Arrays. https://www.me.psu.edu/cimbala/me345/Lectures/Taguchi_orthogonal_arrays.pdf.

[20] Goyal S., Parashar A. (2018). Machine learning application to improve COCOMO model using neural networks. Int. J. Inf. Technol. Comput. Sci..

[21] Hoseinzadeh S., Sohani A., Ashrafi T.G. (2021). An artificial intelligence-based prediction way to describe flowing a Newtonian liquid/gas on a permeable flat surface. J. Therm. Anal. Calorim..

[22] Sohani A., Hoseinzadeh S., Samiezadeh S., Verhaert I. (2021). Machine learning prediction approach for dynamic performance modeling of an enhanced solar still desalination system. J. Therm. Anal. Calorim..

[23] Rankovic D., Rankovic N., Ivanovic M., Lazic L. (2021). Convergence rate of Artificial Neural Networks for estimation in software development projects. Inf. Softw. Technol..

[24] Suresh K., Dillibabu R. (2020). A novel fuzzy mechanism for risk assessment in software projects. Soft Comput..

[25] Hall Elaine M. (1998). Managing Risk: Methods for Software Systems Development.

[26] Pressman R.S. (2001). Software Engineering—A Practitioner’s Approach.

[27] Iranmanesh S.H., Khodadadi S.B., Taheri S. Risk assessment of software projects using fuzzy inference system. Proceedings of the 2009 International Conference on Computers & Industrial Engineering IEEE.

[28] Madachy R. (1997). Heuristic Risk Assessment Using Cost Factors. IEEE Softw..

[29] Odzaly E.E., Greer D., Sage P. Software Risk Management Barriers: Empirical Study. Proceedings of the 3rd International Symposium on Empirical Software Engineering and Measurement.

[30] Kaur I., Narula G.S., Wason R., Jain V., Baliyan A. (2018). Neuro fuzzy-COCOMO II model for software cost estimation. Int. J. Inf. Technol..

[31] Subasri R., Meenakumari R., Panchal H., Suresh M., Priya V., Ashokkumar R., Sadasivuni K.K. (2020). Comparison of BPN, RBFN and wavelet neural network in induction motor modelling for speed estimation. Int. J. Ambient. Energy.

[32] Mukherjee S., Malu R.K. Optimization of project effort estimate using neural network. Proceedings of the 2014 IEEE International Conference on Advanced Communications, Control and Computing Technologies.

[33] Apolo-Apolo O.E. (2020). A mixed data-based deep neural network to estimate leaf area index in wheat breeding trials. Agronomy.

[34] Pandey M., Litoriya R., Pandey P. (2020). Validation of existing software effort estimation techniques in context with mo-bile software applications. Wirel. Pers. Commun..

[35] Pandey M., Litoriya R., Pandey P. (2020). Applicability of Machine Learning Methods on Mobile App Effort Estimation: Validation and Performance Evaluation. Int. J. Softw. Eng. Knowl. Eng..

[36] Ali A., Gravino C. (2021). Improving software effort estimation using bio-inspired algorithms to select relevant features: An empirical study. Sci. Comput. Program..

[37] Kumar P.S., Behera H.S., Nayak J., Naik B. (2021). A pragmatic ensemble learning approach for effective software effort estimation. Innov. Syst. Softw. Eng..

[38] Zhao H., Zhang C. (2020). An online-learning-based evolutionary many-objective algorithm. Inf. Sci..

[39] Dulebenets M.A. (2020). An Adaptive Island Evolutionary Algorithm for the berth scheduling problem. Memetic Comput..

[40] Liu Z.Z., Wang Y., Huang P.Q. (2020). AnD: A many-objective evolutionary algorithm with angle-based selection and shift-based density estimation. Inf. Sci..

[41] Pasha J., Dulebenets M.A., Kavoosi M., Abioye O.F., Wang H., Guo W. (2020). An Optimization Model and Solution Algorithms for the Vehicle Routing Problem with a “Factory-in-a-Box”. IEEE Access.

[42] D’Angelo G., Pilla R., Tascini C., Rampone S. (2019). A proposal for distinguishing between bacterial and viral meningitis using genetic programming and decision trees. Soft Comput..

[43] Panda N., Majhi S.K. (2020). How effective is the salp swarm algorithm in data classification. Computational Intelligence in Pattern Recognition.

[44] Hastings T., Sajeev A. (2001). A vector-based approach to software size measurement and effort estimation. IEEE Trans. Softw. Eng..

[45] Phannachitta P. (2020). On an optimal analogy-based software effort estimation. Inf. Softw. Technol..

[46] Shukla S., Kumar S. Applicability of Neural Network Based Models for Software Effort Estimation. Proceedings of the 2019 IEEE World Congress on Services (SERVICES).

[47] Promise Software Engineering Repository. http://promise.site.uottawa.ca/SERepository/datasets-page.html.

[48] Chhabra S., Singh H. (2020). Optimizing Design of Fuzzy Model for Software Cost Estimation Using Particle Swarm Optimization Algorithm. Int. J. Comput. Intell. Appl..

[49] Kataev M., Bulysheva L., Xu L., Ekhlakov Y., Permyakova N., Jovanovic V. (2020). Fuzzy model estimation of the risk factors impact on the target of promotion of the software product. Enterp. Inf. Syst..

[50] Zhang L., Lu D., Wang X. (2020). Measuring and testing interdependence among random vectors based on Spearman’s *ρ* and Kendall’s *τ*. Comput. Stat..

[51] Fu T., Tang X., Cai Z., Zuo Y., Tang Y., Zhao X. (2020). Correlation research of phase angle variation and coating performance by means of Pearson’s correlation coefficient. Prog. Org. Coat..

[52] Manali P., Rajib M., Ratnam J.V., Masami N., Behera S.K. (2020). Long-lead Prediction of ENSO Modoki Index using Machine Learning algorithms. Sci. Rep..

[53] Liang H., Zou J., Li Z., Khan M.J., Lu Y. (2019). Dynamic evaluation of drilling leakage risk based on fuzzy theory and PSO-SVR algorithm. Future Gener. Comput. Syst..

